# A Multiple-Well Framework for Human Perceptual Decision-Making

**DOI:** 10.3390/e28020232

**Published:** 2026-02-16

**Authors:** Joseph Fluegemann, Jiaqi Huang, Morgan Lena Rosendahl, Jerome Busemeyer, Jonathan D. Cohen

**Affiliations:** 1 Princeton Neuroscience Institute, Princeton University, Princeton, NJ 08540, USA; 2 Department of Cognitive Science, Indiana University Bloomington, Bloomington, IN 47405, USAjbusemey@iu.edu (J.B.); 3 Mathematica Research Services, Princeton, NJ 08543-2393, USA; 4 Department of Psychology, Princeton University, Princeton, NJ 08540, USA

**Keywords:** quantum cognition, drift diffusion models, perceptual decision making, arousal, cognitive control

## Abstract

We present a quantum cognitive model that integrates the influence of cognitive control into human perceptual decision-making. The model employs a multiple-square-well potential, where each well corresponds to a distinct decision outcome. In this framework, well depth encodes signal strength, while well width represents the domain generality of the outcome. The probability of particle localization within each well determines the subjective probability, which subsequently drives a standard Markovian evidence accumulation process to predict empirical choice and response times. We validate the model using the classic dot motion two-alternative forced-choice (2AFC) task. The model successfully replicates key empirical findings of the task, such as the correlation between motion coherence and drift rates. Furthermore, we apply the model to the Yerkes–Dodson law, capturing the approximate inverted U-shaped relationship between task accuracy and cognitive arousal. We compare two theoretical approaches to modeling arousal (1) as eigenenergy values and (2) as kinetic energy terms, contrasting their qualitative predictions regarding the Yerkes–Dodson law. Our work provides the first quantitative model of arousal’s influence on human perceptual decision-making and establishes a foundation for determining the exact functional form of the Yerkes–Dodson law.

## 1. Introduction

Human perceptual decision-making is the process by which we accumulate and integrate stochastic sensory signals to reach a coherent decision. Traditionally, this process is characterized by the Drift Diffusion Model (DDM), which posits that decision-making arises from the sequential sampling of information until a predefined evidence threshold is reached [1,2] (a list of abbreviations is provided at the end of this document, just before the appendices). DDMs have been applied with remarkable success to various paradigms, including random dot motion (RDM) [2,3] and brightness discrimination tasks [4,5], providing unparalleled predictive accuracy for both choice and response time distributions.

Despite its robustness, the DDM framework has not been well integrated with cognitive control, which describes top-down modulation in perceptual decision-making [6]. While several extensions have attempted to bridge this gap [7,8,9], a critical construct remains poorly understood: arousal. Arousal refers to the level of physiological and psychological alertness and wakefulness [10,11]. For instance, the palpitations and racing thoughts during a high-stakes public speaking event reflect hyper-arousal, whereas the mental fog and slowed reflexes during a long, nocturnal highway drive reflect hypo-arousal. Empirical evidence suggests that arousal is a primary driver of cognitive control [12,13,14]; however, standard DDM models struggle to mechanistically incorporate the Yerkes–Dodson law, a cornerstone of experimental psychology which posits that task performance follows an inverted U-shaped function relative to arousal levels [15]. To date, a quantitative framework that maps these fluctuations of arousal onto the parameters of DDMs remains elusive.

In this work, we propose a novel quantum cognitive model to integrate arousal and cognitive control into the DDM framework. This approach draws from the emerging field of quantum cognition [16,17], which employs the mathematical formalism of quantum probability to explain “irrational” human behaviors without assuming the brain is a physically quantum-mechanical system. This framework has successfully modeled complex cognitive phenomena such as the conjunction fallacy [18,19] and order effects [20]. We argue that quantum cognitive models offer three distinct advantages for modeling perceptual decisions. First, uncertainty in quantum models is an emergent property of operator measurements, whereas classical models often treat variability as an exogenous “noise” parameter. Second, the quantum formalism provides a natural representation of arousal as the “energy” of the system, which aligns with neuroscientific findings linking arousal to the “gain” of the locus coeruleus–norepinephrine (LC-NE) system [21]. Finally, quantum models are fully compatible with existing DDM frameworks; the quantum probability of a state can be mapped to the drift rate of a DDM via mechanisms such as the quantum sequential sampler [19].

Our model employs the quantum dynamics of a multiple square well potential [22,23]. In this framework, each potential well represents a choice, where the well’s depth and width correspond to coherence of the stimulus and domain generality, respectively. We model arousal through two distinct lenses: as the energy eigenvalue of the Hamiltonian and as the kinetic energy of the system. We demonstrate that this model replicates empirical findings in Two-Alternative Forced Choice (2AFC) tasks and, crucially, provides a mechanistic derivation of the Yerkes–Dodson law. The remainder of this paper is organized as follows: Section 2 introduces the relevant background of our work including 2AFC, DDMs, and the Yerkes–Dodson law; Section 3 details our multiple-well framework; Section 4 presents the results of fitting the model to human empirical data; and Section 5 compares our two representations of arousal and discusses their respective predictions regarding the Yerkes–Dodson law.

## 2. Background

### 2.1. Drift Diffusion Model of Two-Alternative Forced Choice Task

In this work, we aim to explain how cognitive control and arousal influence perceptual decision-making within a standard experimental paradigm in psychology: the two-alternative forced-choice (2AFC) task. In a 2AFC task, participants are presented with two options and must select one of them on each trial. A large body of work has shown that behavior in 2AFC tasks is well captured by drift–diffusion models (DDMs) [1,4]. In their simplest form, DDMs describe the evolution of a decision variable (*x*) in time (*t*) as a stochastic differential equation,

(1) $$dx=A\;dt+d^{2}\;dW,$$

where dW denotes Gaussian white noise with mean 0 and variance $d^{2}$, and *A* is a constant known as the mean drift rate. Conceptually, the drift rate *A* reflects the average rate at which evidence accumulates in favor of the correct option, while $d^{2}$ controls the level of noise in the accumulation process and therefore the signal-to-noise ratio.

**Figure 1:**
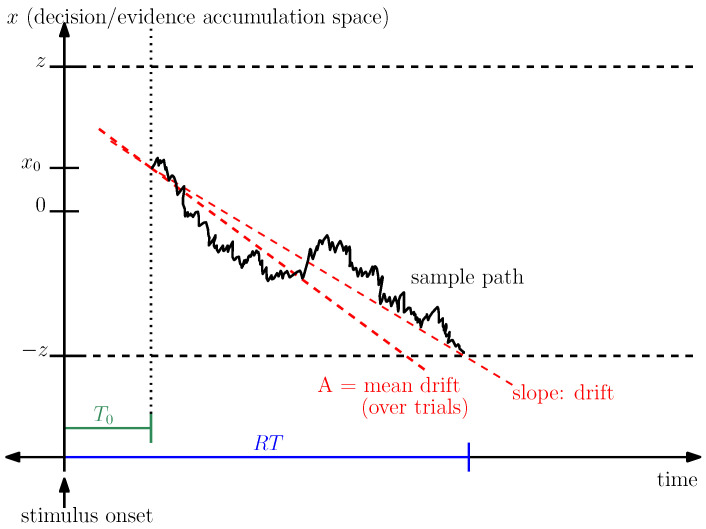
A pictorial illustration of the drift diffusion model. Decision-making is described as a sample path of a drift diffusion process, with mean drift rate A. The decision is made once this sample path reaches one of the two boundaries at *z* and −z, starting from $x_{0}$. $T_{0}$ is the non-decision time, and RT represents the total response time.

Decisions in a DDM are determined by absorbing boundaries that the diffusion process eventually reaches. In a 2AFC task, the two alternatives are typically represented by a symmetric pair of boundaries, located at +z and −z (see Figure 1). The decision process is a sample path of the diffusion starting from an initial state $x_{0}$, which evolves stochastically until it hits one of the two boundaries, thereby determining the choice. Response time (RT) is defined as the elapsed time between stimulus onset and the moment the process reaches a boundary. More precisely, RT is the sum of the decision time (DT) and a non-decision time component $T_{0}$, which captures sensory encoding and motor execution processes that are not directly related to evidence accumulation. That is, $RT=T_{0}+DT$.

**Figure 2:**
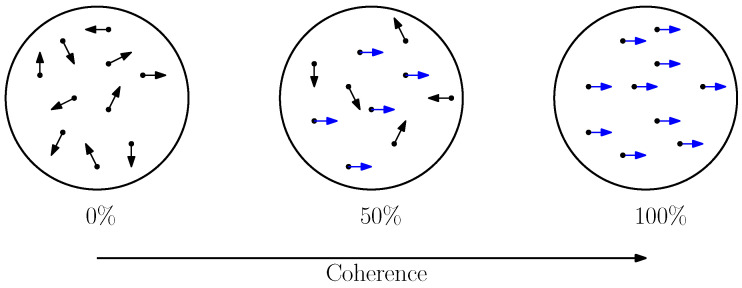
Visualization of the Random Dot Motion task. In this task, human subjects judge the direction of global movement of dots across varying levels of coherence. Coherence represents the ratio of signal dots moving toward a target direction (blue) versus noise dots moving randomly (black).

Although many experimental instantiations of the 2AFC paradigm exist, we focus here on the random dot motion task, as it is one of the most widely used paradigms in perceptual decision-making [3,4,24]. In this task, participants view a field of moving dots on a screen. A subset of the dots moves coherently in a single direction, typically left or right, while the remaining dots move randomly to introduce noise and increase task difficulty. On each trial, participants indicate the perceived direction of coherent motion, after which a new trial begins. A schematic illustration of the random dot motion stimulus is shown in Figure 2. Note that the DDM is describing the participants’ perception of the dot (formalized as evidence accumulation in 1D space for one of two choices); this is distinct from the task itself, of which there are many types—we have chosen the task of dots moving in two dimensions as an example.

More specifically, we focus on behavioral effects reported by Balci et al. [24]. In their experiment, participants viewed dots presented within a 3-inch diameter aperture centered on the screen, with each dot rendered as a 2×2 pixel square. On each trial, a fraction of the dots moved coherently either to the left or to the right, while the remaining dots moved randomly. The direction of coherent motion varied randomly across trials. The ratio of coherently moving dots to randomly moving dots defines the signal-to-noise ratio (SNR), often referred to as motion coherence. Balci et al. manipulated coherence across five levels: 0%, 4%, 8%, 16%, and 32%.

A key finding of Balci et al. [24] is that participants’ estimated mean drift rate *A* scales approximately linearly with motion coherence, as does their level of attentional control. Higher coherence levels make the task easier, facilitate attentional control, and lead to larger drift rates. This systematic relationship is illustrated in Figure 1. An important goal of the present work is to replicate this empirical pattern and to fit the proposed model to human choice and response time data across different coherence conditions. Successfully reproducing this relationship serves as a critical sanity check for the validity of the model and its interpretation in terms of cognitive control and arousal.

### 2.2. Arousal and Yerkes–Dodson Law

**Figure 3:**
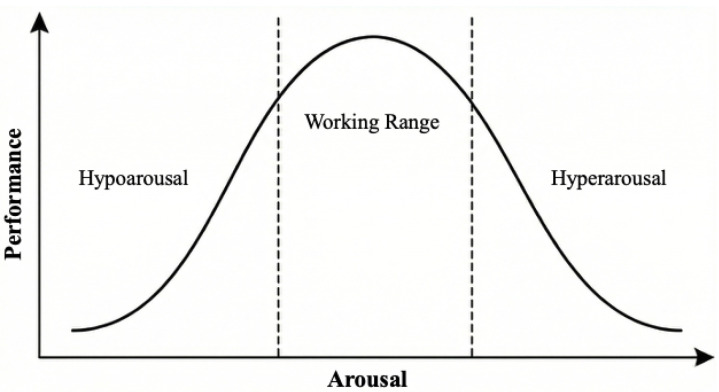
The Yerkes–Dodson Law [15]. This law posits that the relationship between arousal and cognitive performance follows an inverted U-shaped function. Peak performance is achieved at an “optimal” level of arousal (working range); performance declines when an individual is either hypoaroused (insufficiently alert) or hyperaroused (excessively anxious).

Another key aspect that our model seeks to address in perceptual decision-making is the influence of arousal on cognitive performance. A central empirical regularity in this literature is the Yerkes–Dodson law, which states that performance on cognitive tasks, often measured in terms of accuracy or response time (with faster responses indicating better performance), exhibits an inverted U-shaped relationship with arousal (see Figure 3). Although the precise functional form of this relationship remains debated, there is broad agreement that performance is optimal within an intermediate range of arousal. This region is often referred to as the “working range” of arousal. When arousal falls below this range (hypoarousal), performance deteriorates due to insufficient engagement or alertness, whereas excessively high arousal (hyperarousal) is associated with impaired performance, often attributed to increased noise, distractibility, or loss of cognitive control.

In neuroscience, arousal is closely linked to the efficiency of cognitive control processes and is strongly associated with activity in the locus coeruleus–norepinephrine (LC–NE) system. Ref. [21] emphasizes that neuromodulatory systems, particularly the LC–NE system, regulate the “gain” or responsiveness of neural populations. Increased arousal corresponds to higher neural gain, amplifying task-relevant signals but also potentially amplifying noise. Within an intermediate range, this gain modulation enhances the stability and effectiveness of top-down control signals, thereby improving task performance. Importantly, ref. [21]’s framework treats gain modulation as an energetic or resource-like quantity that shapes the dynamics of neural processing rather than as a task-specific control signal. Conceptually, this aligns with interpretations of arousal as a global control parameter that adjusts the overall “energy” available to cognitive systems. Motivated by this perspective, we model arousal as an energy-like quantity within a quantum dynamical framework. The formal structure of this model, and its implications for perceptual decision making, are developed in the next section.

## 3. The Multiple-Particle Multiple-Well Framework for Perceptual Decision-Making

In this section, we introduce and explain our novel quantum multiple-particle multiple-well framework for modeling the 2AFC task. This section’s layout is as follows. We first describe the Hamiltonian of our problem, including the potential and kinetic energy portion, and explain how we obtain choice probabilities in this model. Next, in the last two subsections, we describe two different methods for obtaining the quantum state and modeling arousal: (A) Eigenstate Method and (B) Time-Evolution Method. Finally, we discuss how this quantum framework can be integrated with DDMs to predict empirical choice and response time distributions.

The motivation for using a Hamiltonian-based physics model for understanding decision-making comes from the intuitive notion of decision as a process of settling on a choice, and arousal as some kind of cognitive energy, which we want to combine within a framework of settling within an energy landscape. The energy landscape is provided by the potential energy function, which is the main component of the Hamiltonian. Settling in a local minimum is reminiscent of other work in cognitive science involving a dynamic process of moving towards stable positions in an energy landscape, such as Hopfield networks [25] and cognitive control as in the stability-flexibility tradeoff [26]; these avenues of research conducted the analysis from a classical perspective, which we want to extend to the quantum realm. The quantum approach has inherent stochasticity, and several other advantages mentioned in the general discussion. Since this work considers the 2AFC case of two choices, we model using a potential energy function with two square wells.

### 3.1. General Framework

The dynamics of a quantum system are governed by a Hamiltonian operator and evolve according to the Schrödinger equation,

(2) $$-i\hbar \frac{d}{dt}\psi \left(x,t\right)=\hat{H}\psi \left(x,t\right),$$

where ψ(x,t) is the wavefunction, interpreted as a probability amplitude over position. The Hamiltonian is given by

(3) $$\hat{H}=-\frac{\hbar^{2}}{2m}\frac{\partial^{2}}{\partial x^{2}}+\hat{V}\left(x\right),$$

consisting of a kinetic energy term $-\frac{\hbar^{2}}{2m}\frac{\partial^{2}}{\partial x^{2}}$ and a potential energy term $\hat{V}\left(x\right)$.

**Figure 4:**
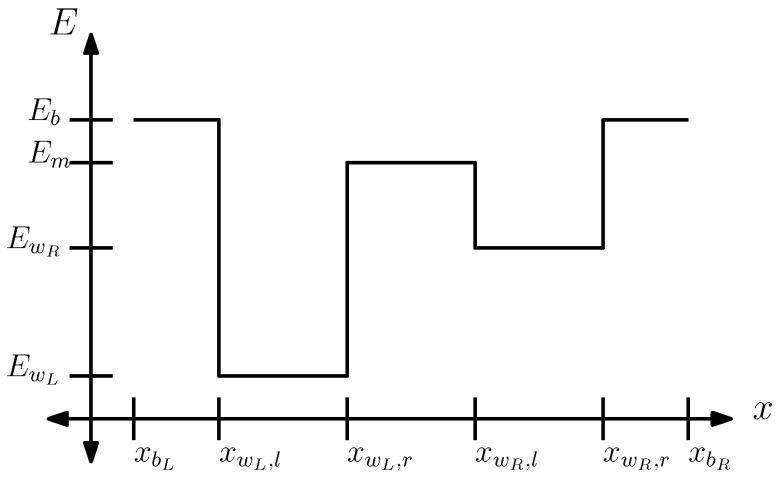
Schematic illustration of the multi-well potential used to model 2AFC decision-making. The two choices are represented by the left and right wells, denoted $w_{L}$ and $w_{R}$. The points $x_{w_{R},l}$ and $x_{w_{R},r}$ indicate the left and right boundaries of the right well, with analogous notation for the left well. The central region between the wells has energy $E_{m}$, which represents the conceptual “gap” between the two choices. The potential satisfies the condition that the two well widths and boundary widths are the same, specifically: $x_{w_{L},l}-x_{b_{L}}=x_{b_{R}}-x_{w_{R},r}$ and $x_{w_{L},r}-x_{w_{L},l}=x_{w_{R},r}-x_{w_{R},l}$. The leftmost and rightmost regions correspond to “free” energy $E_{b}$, located at positions $x_{b_{L}}$ and $x_{b_{R}}$.

To fully specify the quantum dynamics, we begin by defining the form of the potential energy function $\hat{V}\left(x\right)$. Figure 4 illustrates the potential we use to model two-alternative forced-choice (2AFC) decision-making. The potential V(x) is composed of two wells separated by a middle region and bounded by high-energy barriers. A central interpretive assumption of this work concerns the role of the potential function V(x). Specifically, we interpret the two wells of the potential as corresponding to the two alternatives in a 2AFC task. The manner in which choice probabilities are extracted from the quantum dynamics induced by this potential is described in detail in the following section.

Conceptually, the width of the wells corresponds to the semantic range of the two concepts, while the separation between the wells corresponds to how different they are. The height of the wells is related to the attention, coming from the salience of the signal (i.e., signal to noise ratio). Here, we briefly note that in the classical DDM, there is a notion of “attentional control” capturing how easy it is to focus on a task, which is often treated as co-determining the drift rate, along with coherence; in our present treatment, for simplicity, we focused on the role of well depth in modeling the effects of coherence.

To reduce computational complexity, we discretize the position space and represent both the Hamiltonian and the potential energy as matrices. Under discretization, the potential operator takes the diagonal matrix form

(4) $$\hat{V}\left(x\right)=\left[\begin{array}{cccc} V(x_{1}) & 0 & 0 & \cdots \\ 0 & V(x_{2}) & 0 & \vdots \\ \vdots & \ddots & \ddots & \vdots \\ 0 & \cdots & 0 & V(x_{n}) \end{array}\right],$$

where $x_{1},\ldots ,x_{n}$ denote the discretized position points.

We similarly discretize the kinetic energy operator. We use a common method called the Matrix Numerov method [27]. This method is particularly effective for finding bounded eigenstates in well potentials. The Matrix Numerov method provides a candidate matrix *K* for the discretization of the kinetic energy operator which will be described in more detail in Appendix A.

We multiply the matrix *K* above by a constant α that represents the magnitude of the kinetic energy. This parameter α will scale the expectation value of the kinetic energy, giving us freedom to increase or decrease this energy in relation to that of the potential energy. In summary, we have the following expression for the matrix representation of the Hamiltonian:

(5) $$\hat{H}:=\alpha K+\hat{V}\left(x\right).$$

#### Connection to DDM

Because each potential well represents one alternative in a 2AFC task, we define the quantum probability of making a particular choice as the probability of finding the particle within the corresponding well. Let us denote the correct choice of well by a label *C*; thus, if the correct choice is the right well, then C=R, and otherwise C=L. The probability that the particle finds the correct well is then given by

(6) $$P_{C}=\int_{x_{w_{C},l}}^{x_{w_{C},r}}{\left|\psi \left(x,t\right)\right|}^{2}\;dx.$$

We refer to this quantity as the mean integration efficiency (MIE) for the correct option. Intuitively, it measures the extent to which quantum probability mass accumulates in the region associated with the correct choice.

For the alternative (incorrect) option (denoted by *I*), corresponding to the other well, we define the MIE as

(7) $$P_{W}=\int_{x_{w_{I},l}}^{x_{w_{I},r}}{\left|\psi \left(x,t\right)\right|}^{2}\;dx.$$

Because the particle may also occupy regions outside the wells (i.e., classically forbidden or undecided regions), the probabilities of finding the particle in the two wells do not, in general, sum to one. Indeed, these integrals are not intended to represent choice probabilities directly, and are instead treated as the mean integration efficiency that informs the drift rate of a standard drift diffusion process, where the final predicted probabilities do sum to 1.

Recall that the mean drift rate *A* in the drift-diffusion model (DDM) quantifies the net tendency for evidence to accumulate in favor of the correct choice rather than the incorrect one. We relate this mean drift rate to the two MIEs via

(8) $$A=\lambda \;(P_{C}-P_{W}),$$

where λ is a scaling parameter, typically chosen to match the maximum attainable magnitude of the mean drift rate. This construction is inspired by the definition of drift rates in the Quantum Sequential Sampler framework [19]. With $d^{2}$ treated as a free diffusion parameter, standard DDM methods can then be used to derive the predicted choice probabilities and response time distributions.

We summarize our three main parameters and their effects on the drift rate, alongside their cognitive interpretations in Table 1.

With the Hamiltonian (V(x)) and the state (ψ(x,t)) defined, we possess the data required to derive choice probabilities and drift rates. Having already addressed the Hamiltonian, we will now focus on the formulation of ψ(x,t), which can be defined with two different methods.

### 3.2. Method (A): Eigenstate Method, Also Classical-Quantum Method, or Total Energy Arousal Model

Our first method is the one adapted from [22,28]. Here, ψ(x,t) is time independent and an eigenstate of the Hamiltonian. To obtain this eigenstate, the model requires two part: in addition to the Hamiltonian $\hat{H}$ described above, representing the quantum part, this model also requires a classical probability distribution over energies.

First, we can take $\hat{H}$ and solve the time-independent Schrodinger equation for the bounded energy eigenstates $\left\{\psi_{0},\psi_{1},\ldots ,\psi_{i},\ldots ,\psi_{f}\right\}$ (*f* for final) with corresponding eigenvalues $E_{0},E_{1},\ldots ,E_{i},\ldots ,E_{f}<E_{b}$, where $E_{b}$ is the free energy (the top of the wells). $E_{0}$ is the lowest energy (corresponding to the ground state $\psi_{0}$), and $E_{f}$ is the highest energy bound state with energy less than $E_{b}$.

**Figure 5:**
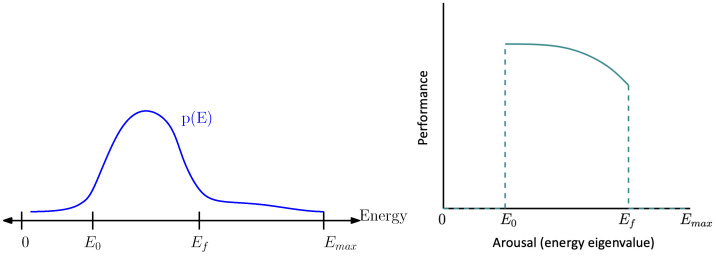
(**Left**): Sampling distribution of energies to model arousal. When the sampled energy is not within the range $\left[E_{0},E_{f}\right]$, the model assume that people are not optimally aroused. (**Right**): Method (A)’s prediction of the Yerkes-Dodson law. Performance is effectively zero in hypoarousal $\left(0,E_{0}\right)$ and hyperarousal ranges $\left(E_{f},E_{max}\right]$, with a decreasing trend within $\left[E_{0},E_{f}\right]$.

Simultaneously, we define a classical “sampling” distribution p(E) over continuous energies in $\left(0,E_{max}\right]$, where $E_{max}$ is some maximum energy allowed in the cognitive system. Figure 5 illustrates this distribution. Note that since *E* is a continuous random variable, *E* does not have to be one of the eigenvalues, and can be less than $E_{0}$.

Given these two parts, the process of obtaining the state ψ(x,t) is as follows. (1) We sample an energy from the probability distribution p(E), denoted as $E^{*}$, and define a range of energy $\left[E^{*},E^{*}+\delta \right]$, where δ corresponds to the maximum gap between two arbitrary eigen-energies; (2) We then randomly select an eigen-energy that falls within this range (drawn uniformly), denoted as $E_{i}^{*}$. The eigenstate corresponds to $E_{i}^{*}$ will then be selected as ψ(x,t).

Since the existence of $E^{*}$ is critical for the functioning of the model, it is natural to interpret $E^{*}$ as a criterion for optimal arousal in a given perceptual process. Note that because δ is the maximum spectral gap between adjacent eigenvalues, for $E_{0}\leq E^{*}\leq E_{f}$, the spectral structure guarantees the existence of at least one eigenstate. If no bounded eigenstate exists in the admissible range and $E^{*}<0$, the model predicts hypoarousal. Conversely, if $E^{*}>E_{f}$, the model predicts hyperarousal, indicating that the system is driven beyond the highest energetically supported perceptual state.

In our model, the MIE for both wells will be undefined in the case when $E^{*}$ falls outside of $\left[E_{0},E_{f}\right]$. In this case, we will simply say that the DDM model is undefined and there is zero probability of obtaining either the correct or the wrong choices; thus, the accuracy (performance) is zero. When $E^{*}$ falls within $\left[E_{0},E_{f}\right]$, performance decreases as a function of $E_{*}$, assuming the well corresponds to the correct option is always deeper.

Given these, the shape of the Yerkes–Dodson law predicted by this model is expected to be that shown in Figure 5. Note that this qualitatively replicates the expected inverted u-shaped-like pattern found in [15]’s empirical data; however, it predicts sharp drops at the two endpoints at $E_{0}$ and $E_{f}$. Whether these sharp drops are empirically accurate remains debatable [21], but a smoother-shaped version of the model can also be formulated within this framework. In the next section, we introduce this smoother version of the model.

### 3.3. Method (B): Time-Evolution Method, Also Quantum Method, or Kinetic Energy Arousal Model

Our second method was meant to address some of the weakness of the first approach. Here, ψ(x,t) is a state that is obtained by unitary time evolution from an initial state. Thus, the description is completely quantum, beginning with a quantum state and ending with a time-evolved state.

Being completely quantum, this method lends itself naturally to the interpretation of arousal as the magnitude of the kinetic energy (that is, the magnitude of α that scales the Matrix Numerov kinetic term). Besides, this method produces smooth performance versus arousal curves that more closely resemble the theoretical inverted-U-shaped curve of the Yerkes–Dodson law (see Figure 8 for illustrations). Finally, methods (A) and (B) yield distinct predictions regarding the functional form of the Yerkes–Dodson law. This provides an opportunity to empirically address the law’s precise shape, which remains a subject of ongoing debate.

To obtain ψ(x,t), we begin by defining the initial state of the quantum dynamics as a uniform superposition:

(9) $$\psi \left(x,0\right)=\left\{\begin{array}{cc} \frac{1}{\sqrt{x_{b_{R}}\;-\;x_{b_{R}}}} & x_{b_{L}}<x<x_{b_{R}}\\ 0 & \mathrm{otherwise}. \end{array}\right.$$

When discretized, ψ(x,0) becomes the vector: $\psi_{0}\left(x\right)={\left(\frac{1}{\sqrt{n}},\frac{1}{\sqrt{n}},\ldots ,\frac{1}{\sqrt{n}}\right)}^{T}$, for dimension *n*. We choose this state because it is the simplest initial state that is neutral with respect to the choice probabilities, with equal probability to be found in either well at the start.

Since the Hamiltonian is time-independent, the solution of the Schrodinger’s equation for computing ψ(x,t) can be written as

(10) $$\psi \left(x,t\right)=e^{-iHt}\psi \left(x,0\right).$$

Recall that the response time in a 2AFC task is commonly decomposed into a residual latency component $T_{0}$ and a decision time DT, such that $RT=T_{0}+DT$ [7]. Within the present modeling framework, the DDM accounts only for the decision time DT. The non-decision time $T_{0}$ is instead associated with the quantum time evolution that generates the state $\psi (x,T_{0})$, which we will use to integrate with DDM. We emphasize that our work does not replace the DDM, since we do not actually calculate DT; rather, we provide a mechanism describing what happens during $T_{0}$. The result is a probability distribution, but the participant must sample an evidence accumulation process like the DDM to understand what its shape is.

This treatment differs from the static choice of ψ(x) in method (A), where the eigenstates are assumed to be fixed and the DDM implicitly accounts for both $T_{0}$ and DT. From this perspective, our model provides an explicit theoretical account of non-decision time in the DDM, which is often treated as a fixed constant, typically between 0.2 and 0.5 s and independent of the model dynamics [4].

## 4. Empirical Test of the Model

### 4.1. Fitting to Mean Drift Rates

To validate our model, we first examine its ability to explain traditional perceptual decision-making findings via DDMs. This establishes a baseline for our approach within this field. In particular, we test whether our multiple-particle multiple-well framework, which predicts DDM mean drift rates, can improve model parsimony by reducing free parameters without sacrificing goodness-of-fit significantly.

The experimental data for our model fitting comes from the dot motion task in [24]. We aim to replicate the 85 mean drift rates (across five coherence levels and 17 participants) using the multiple-particle, multiple-well framework.

For now, we prioritize method (B) as a starting point for its simplicity. Method (A) requires a more complex definition of p(E) compared to the uniform initial state of method (B), which would increase the number of free parameters. Furthermore, method (B) is computationally more efficient, as it avoids repeated eigenstate calculations by scaling the Hamiltonian’s off-diagonal elements by a constant α. Although method (A) is viable, it is left for future research.

Specifically, we use a total of 28 parameters to accommodate the 85 mean drift rates that were originally estimated as free parameters in [24]. The following is a summary of these parameters:

- We fixed $E_{b}$ at 1 and fit a single $E_{w_{L}}$ and $E_{m}$ across all coherence conditions and participants (2 parameters). This establishes the energy scale for the fitting process.
- We let $E_{w_{R}}$ vary across the five coherence conditions but kept it constant across participants (5 parameters). This accounts for varying attentional control over the five different levels of motion coherence.
- We fit a well width for the left well and a width for the left edge ($x_{w_{L},l}-x_{b_{L}}$ in Figure 4), setting these equal to the right well and right edge widths, respectively. Since width represents the generality of a concept, maintaining symmetry assumes that “dot moving left” and “dot moving right” are equivalent concepts that do not differ in generality (2 parameters).
- We fit a single time $T_{0}$ for the time evolution, corresponding to the non-decision time, across all conditions and participants (1 parameter). Although a simplification, fixing a single non-decision time is a common convention in the DDM literature [4].
- We fit a single scaling constant λ across all participants and coherence conditions (1 parameter).
- For each participant, we fit a single scaling constant α across all coherence conditions (17 parameters). This assumes that individual differences arise from the varying arousal levels of the participants.

Conceptually, with this parameterization setup, we assume that the experimental stimuli and environment define the potential landscape of the Hamiltonian, while individual differences in arousal define the kinetic energy. For a fixed environment (coherence), we assume that the landscape remains unchanged across participants. Across different coherence conditions, we assume that some tasks are harder than others, reflected in the varying depth of the correct well, where deeper wells correspond to easier tasks. However, because an optimal arousal level exists, individual differences in performance are driven by participant-specific arousal levels, which are modeled by allowing α to vary across individuals.

We used the particleswarm function in MATLAB v. 25.2.0 and fit the model by minimizing the squared error between the fitted mean drift rates reported in [24] and the predictions of our model. The scripts we wrote are available online in our GitHub v. 3.5.4 repository at the link listed in the Data Availability Statement below.

**Figure 6:**
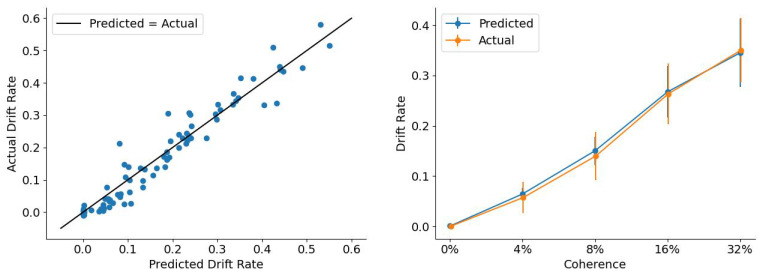
(**Left**): Comparison of empirical drift rates (estimated as free parameters in [24]) against predicted values from the multiple-particle, multiple-well framework. (**Right**): Drift rate as a function of motion coherence, comparing the original data (orange; ref. [24]) with our model predictions (blue).

We achieved a total squared error of 0.121. Figure 6 displays the empirical drift rates against the drift rates predicted by our model. The points closely align with the diagonal line x=y, suggesting a good fit. We then examine whether we can replicate Figure 5a from [24], in which fitted drift rates scale linearly with coherence. As shown in Figure 6, we successfully replicate this major empirical result from [24] using only 28 free parameters instead of 85.

**Figure 7:**
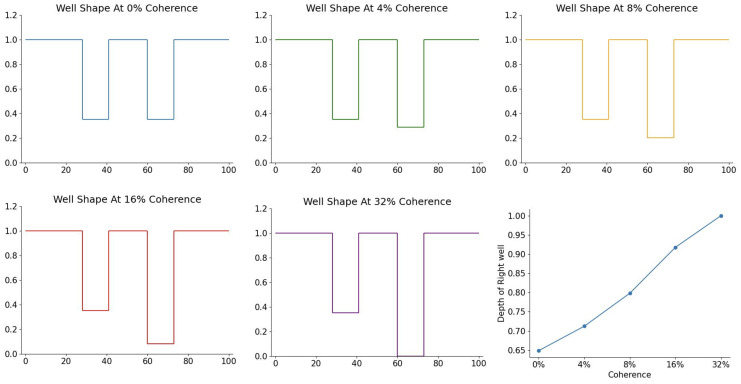
(**First five plots**): Fitted potential energy landscapes for the 5 different coherence levels described in the main text, in order of increasing coherence. (**Last plot**): Depth of the variable (right) well versus coherence; this plots the depths of the right wells against coherence in the previous 5 figures.

This linear relationship is driven by the depth of the “correct” well ($E_{w_{R}}$), which we define as a free parameter varying with coherence. As shown in Figure 7, $E_{w_{R}}$ increases linearly with coherence. A deeper well produces a larger drift rate according to Equation (8), accounting for the coherence-dependent shifts in the model’s decision dynamics.

### 4.2. Prediction of Yerkes–Dodson Law

Another key objective for our model is to capture the qualitative predictions of the Yerkes–Dodson law. We aim to compare the model’s predicted performance-arousal relationship against the classic findings of Yerkes and Dodson, which describe an inverted-U relationship: performance improves with increasing arousal at low levels, reaches an optimal peak, and subsequently declines as arousal continues to rise.

In [24], performance is measured as the accuracy in predicting the dot’s movement direction. This is predicted by the mean drift rate (*A*): a more positive mean drift rate signifies higher performance. Besides, since we utilize method (B) to fit the empirical data, arousal is represented by the scaling constant α, the magnitude of kinetic energy. Consequently, to examine the Yerkes–Dodson law, we examine how the mean drift rate *A* (performance) varies as a function of the scaling constant α (arousal).

**Figure 8:**
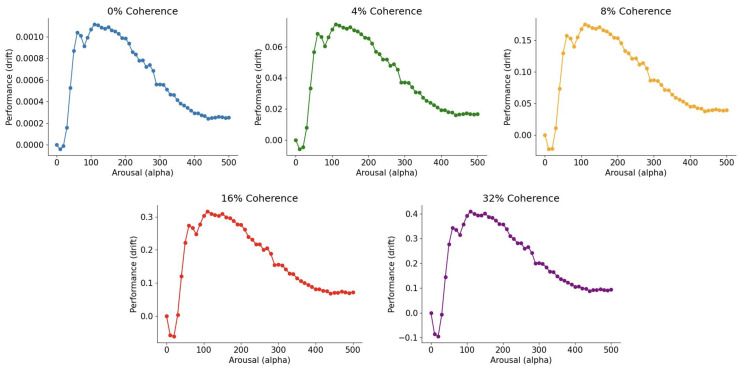
Model-predicted performance as a function of arousal across five coherence conditions, demonstrating the emergent inverted-U relationship of the Yerkes–Dodson law. Performance is operationalized on the y-axis as the mean drift rate *A*, while arousal is represented on the x-axis by the kinetic energy scaling constant α.

Figure 8 illustrates the relationship between *A* and α over the interval [0,200] across five coherence conditions. These plots are generated using the best-fitting parameters, holding factors other than arousal constant; for the time, given that we expect experimentally for participants to have slightly varying (even on a trial-by-trial basis) non-decision times $T_{0}$, we plotted using an average of times in a small interval around the fitted time $T_{0}$. Notably, the curves maintain a consistent inverted-U shape, notwithstanding small oscillations in a few places, with the primary difference across coherence levels being the vertical scaling of the function (e.g., zero coherence has performance generally worse than 32% coherence throughout the entire curve).

Comparing these results to the qualitative shape of the Yerkes–Dodson law derived from method (A) (Figure 5, right), the curves generated here exhibit significantly greater smoothness. Notably, they lack the sharp discontinuities at the physiological extremes of hypo- and hyper-arousal. While the precise quantitative form that best aligns with empirical data remains an open question, both methods offer robust, empirically testable predictions of the Yerkes–Dodson relationship within our modeling framework, providing a clear foundation for future experimental verification.

## 5. General Discussions

In this work, we present a novel framework that integrates cognitive control and arousal into the traditional drift-diffusion model of perceptual decision-making, using the mathematical formalism of quantum theory. Our framework not only successfully replicates major empirical results in perceptual decision-making but also yields novel, empirically testable predictions regarding the relationship between task performance and arousal.

It is important to note that prior several studies have attempted to address the Yerkes–Dodson law within a quantum framework. For instance, ref. [23] proposed a perturbative method that models arousal as energy input into an open quantum system. Their model predicts sustained oscillations, which could provide a valuable point of comparison for the specific form of the Yerkes–Dodson law derived in our current research. Additionally, ref. [29] utilized Lindbladian open quantum system dynamics. We do not provide a direct quantitative comparison with these models here because neither has been fitted to empirical data, nor have they been fully integrated with DDMs to provide joint predictions for choice and response time (RT) distributions, as demonstrated in this paper. Future work can explore how they can be integrated with DDMs.

Regarding the functional form of the Yerkes–Dodson law, existing literature has proposed various shapes ranging from the classic inverted-U to oscillatory patterns [21,30,31,32]. Our objective in this work is not to resolve the debate in this work but rather to propose a new, quantitatively testable prediction regarding the law’s underlying mechanism. We acknowledge the existence of diverse theoretical shapes and encourage future research to further elucidate the exact nature of this relationship.

In addition, we acknowledge that our model’s parameters may not be fully identifiable given the experimental setting in [24]. This limitation arises because the sample size described in [24] is relatively small, consisting of only 17 participants with 85 drift rates. However, despite possible identifiability issues, the primary objective of the present work is not to determine the precise magnitude of these parameters or to prove strict identifiability; rather, we aim to verify whether the model can replicate key empirical findings in perceptual decision-making while incorporating the effect of arousal. Our results demonstrate that we have successfully achieved this goal. Future research can address these identifiability issues by conducting rigorous parameter recovery studies and employing larger sample sizes. Additionally, obtaining an independent measurement of the non-decision time $T_{0}$ would help constrain this variable which interacts with all other parameters in the Hamiltonian, thereby improving overall identifiability.

A key theoretical remark is that our proposed model utilizes a conservative force within a closed quantum system. An alternative approach would be to employ an open quantum system where the dynamics involve friction and dissipation (e.g., Lindbladian dynamics [29,33]). Such dynamics might allow for a more seamless integration with non-conservative diffusion processes. However, we also note that such an open system model requires simulating the dynamics with a density matrix rather than a state vector, which introduces a complexity scaling that squares the dimension of the underlying quantum state space. Additionally, extending our model to higher-dimensions might allow other quantum effects like incompatibility to be studied, but this also scales the size of the Hamiltonian exponentially with dimension. These requirements make numerical simulations computationally demanding, so they cannot currently be scaled to larger sized models given current software and hardware limitations. Therefore, exploring the ways in which quantum computing can provide a computational advantage when dealing with these issues of scalability is another interesting avenue for future research. We also mention here one alternative “barrier model” approach that models decision making by whether an electron can tunnel through a barrier in a potential well [34]. We studied a well model in this work, given the ease with which the cognitive parameters can be connected to our potential function, but note that it would be interesting to compare the different approaches in future work.

As aforementioned, we stress that our work uses the mathematical formalism of quantum physics as a tool to explain human behaviors, without assuming that the brain is physically a quantum-mechanical system. The program is motivated by extensive empirical evidence that human behaviors violate classical axioms of rationality and probability theory, and focuses only on providing computational-level predictions of “quantum-like” effects in human behaviors. Nevertheless one idea for why quantum mathematics would be relevant for cognitive systems is that classical neural networks only allow completely positive or completely negative interference while quantum systems allow a continuous range of interference and interactions among units [35]. In terms of how these quantum-like effects could manifest in the brain, we emphasize that our model has not yet been integrated with neural-level architectures (but please see the next paragraph).

Finally, we conclude by providing several directions to connect our model with neural dynamics. First, because we have specified that well depth corresponds to signal strength (coherence), this could have a neuroscientific formulation in terms of the norm of the vector that encodes the signal via, say, a pattern of activity in a neural population. There have been some works that have addressed this already, e.g., ref. [36], though not specifically using the well model we proposed in this work. Additionally, in the psychological space, we often think of this parameter as also being subject to internal regulation by attentional control, which, in previous work, has also been considered to influence well depth [22]. Furthermore, while we have not addressed the effects of well width in the present work, we note that previous work has addressed this, suggesting that it may be useful in modeling representational structure (e.g., with narrower wells corresponding to more precise concepts and wider ones to more general ones). This might, in turn, be related to the width of attractors and/or representational sharing in neural networks (e.g., [26]). Finally, our work suggests the parameter α can be used to model arousal—a construct that appears frequently in neuroscientific and psychological research, and is commonly assumed to be regulated by neuromodulatory systems (in particular, norepinephrine; NE), but is rarely given a formally rigorous mechanistic interpretation. One potential link is the idea that NE may serve to modulate the gain of neural processing (e.g., [21]), which can be interpreted as inverse-temperature, which in turn might be related to the construct of energy in our framework. This remains intriguing, and potentially an important direction for future work.

## Figures and Tables

**Table 1:** The conceptual meaning of parameters in our multiple particle multiple well model and their connection to the drift rate of the drift diffusion model in Equation (8).

Parameter	Effect on Drift Rate	Cognitive Interpretation
well depth of the “correct” well	deeper = larger $P_{C}$	correct option’s signal strength
well width of the “correct” well	wider = larger $P_{C}$	correct option’s conceptual generality
kinetic energy strength α	there exists optimal α that maximizes $P_{C}-P_{W}$	arousal and gain modulation

## Data Availability

The data and code used in the analysis described in this article can be found at https://github.com/JosephFlueg/a_multiple_well_framework_for_human_perceptual_decision_making, accessed on 4 January 2026.

## Abbreviations

The following abbreviations are used in this manuscript:

2AFC	Two-alternative forced-choice (task).
DDM	Drift diffusion model.
DT	Decision time.
LC-NE	Locus coeruleus–norepinephrine (system).
MIE	Mean integration efficiency.
MPMW	Multiple-particle multiple-well.
NE	Norepinephrine.
RDM	Random dot motion.
RT	Response time
SNR	Signal-to-noise ratio.

## Appendix A. Matrix Numerov Method

In this appendix, we provide an overview of the Matrix Numerov method for computing the discretization of the kinetic energy operator in nonrelativistic quantum mechanics. This is useful for doing calculations involving the Schrodinger equation.

We noted above that the kinetic energy matrix takes the following form:

$$K=-\frac{B^{-1}A}{2}$$

Suppose we discretize our space to have *n* points. The matrices *A* and *B* are as follows:

*B* is an n×n matrix that starts with 10 along the diagonal and 1 along the next-to-diagonals. We divide the entire matrix by 12, so that the diagonal terms are approximately 0.833 and the next-to-diagonals are approximately 0.083:

$$B=\frac{1}{12}\left(\begin{array}{ccccccc} 10 & 1 & 0 & 0 & 0 & 0 & \cdots \\ 1 & 10 & 1 & 0 & 0 & 0 & \cdots \\ 0 & 1 & 10 & 1 & 0 & 0 & \cdots \\ 0 & 0 & 1 & 10 & 1 & 0 & \cdots \\ \vdots & & & \vdots & & & \ddots \end{array}\right)=\left(\begin{array}{ccccccc} 0.833 & 0.083 & 0 & 0 & 0 & 0 & \cdots \\ 0.083 & 0.833 & 0.083 & 0 & 0 & 0 & \cdots \\ 0 & 0.083 & 0.833 & 0.083 & 0 & 0 & \cdots \\ 0 & 0 & 0.083 & 0.833 & 0.083 & 0 & \cdots \\ \vdots & & & \vdots & & \ddots \end{array}\right)$$

*A* is another n×n matrix, starting with −2 along the diagonals and 1 along the next-to-diagonals. The matrix *A* comes from the first-order approximation of the second derivative operator $\frac{\partial^{2}}{\partial x^{2}}$. We can let Δx be the size of the gaps in our discretization and write the derivative

$$\frac{\partial }{\partial x}\psi \left(x\right)=\lim_{\Delta x\to 0}\frac{\psi (x+\Delta x)-\psi (x)}{\Delta x}$$

Applying the derivative again:

$$\frac{\partial }{\partial x}\left(\frac{\partial }{\partial x}\right)=\frac{\partial }{\partial x}\left(\lim_{\Delta x\to 0}\frac{\psi (x+\Delta x)-\psi (x)}{\Delta x}\right)=\lim_{\Delta x\to 0}\frac{\frac{\psi (x+\Delta x)-\psi ([x+\Delta x]-\Delta x)}{\Delta x}-\frac{\psi (x)-\psi (x-\Delta x)}{\Delta x}}{\Delta x}$$

This is:

$$\lim_{\Delta x\to 0}\frac{\psi (x+\Delta x)-2\psi (x)+\psi (x-\Delta x)}{{\left(\Delta x\right)}^{2}}$$

Thus, if we write this as an operator with finite Δx, $\frac{-\hbar^{2}}{2m}\frac{\partial^{2}}{\partial x^{2}}$ becomes

$$-\frac{\hbar^{2}}{2m{\left(\Delta x\right)}^{2}}\left(\begin{array}{ccccc} -2 & 1 & 0 & 0 & \cdots \\ 1 & -2 & 1 & 0 & \vdots \\ \vdots & & \ddots & & \vdots \\ \vdots & 0 & 1 & -2 & 1\\ \cdots & 0 & 0 & 1 & -2 \end{array}\right):=-\frac{\hbar^{2}}{2m{\left(\Delta x\right)}^{2}}M$$

In the Matrix Numerov method, there is a constant, usually denoted δ, that corresponds to the spacing of the discretization. This is essentially the Δx in the equations above.

For example, if $\frac{1}{\delta^{2}}=200$, we would have:

$$A=200(\begin{array}{ccccccc} -2 & 1 & 0 & 0 & 0 & 0 & \cdots \\ 1 & -2 & 1 & 0 & 0 & 0 & \cdots \\ 0 & 1 & -2 & 1 & 0 & 0 & \cdots \\ 0 & 0 & 1 & -2 & 1 & 0 & \cdots \\ \vdots & & & \vdots & & & \ddots \end{array})=(\begin{array}{ccccccc} -400 & 200 & 0 & 0 & 0 & 0 & \cdots \\ 200 & -400 & 200 & 0 & 0 & 0 & \cdots \\ 0 & 200 & -400 & 200 & 0 & 0 & \cdots \\ 0 & 0 & 200 & -400 & 200 & 0 & \cdots \\ \vdots & & & \vdots & & & \ddots \end{array})$$

The matrix *B* operates like a higher-order correction term.

Thus, comparing with the expression $\alpha K=-\alpha \frac{B^{-1}A}{2}$ for the kinetic energy term in the Hamiltonian, we see that α, which represents arousal in our cognitive model, essentially corresponds to $\frac{\hbar^{2}}{m}$ in the equation, and it controls the relative size of the kinetic energy in relation to the potential energy.

For reference, we give the explicit form of $B^{-1}M$ (where $\alpha K=-\alpha \frac{B^{-1}A}{2}=-\alpha \frac{B^{-1}M}{2\delta^{2}}$)

$$B^{-1}M=\left(\begin{array}{cccccc} -2.547 & 1.470 & -0.14854 & 0.014997 & -0.001515 & \\ 1.470 & -2.695 & 1.4854 & -0.14997 & 0.015415 & \\ -0.14854 & 1.4854 & -2.697 & 1.4847 & -0.14999 & \\ 0.014997 & -0.14997 & 1.4847 & -2.697 & 1.4847 & \\ -0.001515 & 0.015415 & -0.14999 & 1.4847 & -2.697 & \\ & & & & & \ddots \end{array}\right)$$

Note the form of this matrix. It is symmetric, both along the diagonal (which is evident from what we have shown) and antidiagonal (which we have not shown, except with ⋱). The main diagonal is negative, and each neighboring diagonal is alternating positive and negative. Each successive diagonal decreases in absolute value by a factor of approximately 10. The values in each diagonal are similar—they stay the same from the third entry onwards. Most of these statements stay true when we multiply by the constant $\frac{-\alpha }{2\delta^{2}}$.

Additionally, by the next argument, we can always add a constant along the diagonal while not affecting the probabilities we calculate. Therefore, it is mostly the immediate off-diagonals, whose magnitude is governed by α, which determine the impact of the kinetic energy on the results of the modeling.

We note here that we can add a constant *c* to the potential V(x) without changing the physics. This is true in general (even when the Hamiltonian is time-dependent), but is particularly easy when *H* is time-independent. In this case, adding a constant *c* to the potential does not add any time-dependence. Thus, denoting the new Hamiltonian $\tilde{H}=H+c$, where *c* is the additional constant, the solution to the Schrodinger equation is: $\psi \left(x,t\right)=e^{-i\tilde{H}t/\hbar }\psi \left(x,0\right)=e^{-ict/\hbar }e^{-iHt/\hbar }\psi \left(x,0\right)$. When we square to obtain probabilities, because the extra factor of $e^{-ict/\hbar }$ is just a complex number of unit norm, it cancels out.

## Appendix B. Optimal Parameter Results of Fitting

Here, we record the actual values of the parameters producing the best fit. This will be displayed in the following tables.

**Table A1:** This table contains the fitted values of the following parameters in the well model: edge width, well width, λ, time, $E_{0}$ and $E_{m}$ the heights of the left and middle wells, respectively.

Edge Width	Well Width	$T_{0}$	λ	$E_{0}$	$E_{m}$
28.0514	13.4231	3.9883	2.4738	0.3522	1.0000

Next, we record the values of the parameters corresponding to the five SNR conditions.

**Table A2:** This table contains the fitted values the heights $E_{w_{R}}$ of the right wells (one for each coherence condition).

$E_{w_{R}}\left(0\%\right)$	$E_{w_{R}}\left(4\%\right)$	$E_{w_{R}}\left(8\%\right)$	$E_{w_{R}}\left(16\%\right)$	$E_{w_{R}}\left(32\%\right)$
0.3512	0.2876	0.2018	0.0823	0.0000

Finally, we show the results of the fitting procedure for the values of α.

**Table A3:** Fitted α values for the 17 participants.

ID	1	2	3	4	5	6	7	8	9
** α **	151.38	55.86	176.80	111.82	60.39	177.01	43.10	54.14	176.09
ID	10	11	12	13	14	15	16	17	
** α **	173.69	54.24	100.86	190.69	57.89	168.80	156.66	119.01	
